# Positive Aspects of Oxidative Stress at Different Levels of the Human Body: A Review

**DOI:** 10.3390/antiox11030572

**Published:** 2022-03-17

**Authors:** George Jîtcă, Bianca E. Ősz, Amelia Tero-Vescan, Amalia Pușcaș Miklos, Carmen-Maria Rusz, Mădălina-Georgiana Bătrînu, Camil E. Vari

**Affiliations:** 1 Department of Pharmacology and Clinical Pharmacy, Faculty of Pharmacy, George Emil Palade University of Medicine, Pharmacy, Science and Technology of Târgu Mureș, 540139 Târgu Mureș, Romania; george.jitca@umfst.ro (G.J.); camil.vari@umfst.ro (C.E.V.); 2 Department of Biochemistry, Faculty of Pharmacy, George Emil Palade University of Medicine, Pharmacy, Science and Technology of Târgu Mureș, 540139 Târgu Mureș, Romania; amelia.tero-vescan@umfst.ro (A.T.-V.); amalia.miklos@umfst.ro (A.P.M.); 3 Doctoral School of Medicine and Pharmacy, I.O.S.U.D, George Emil Palade University of Medicine, Pharmacy, Science and Technology of Târgu Mureș, 540139 Târgu Mureș, Romania; carmenrusz20@gmail.com (C.-M.R.); madalina-georgiana.batrinu@umfst.ro (M.-G.B.)

**Keywords:** oxidative stress, hormesis, neurodegeneration, physical exercise, antioxidants

## Abstract

Oxidative stress is the subject of numerous studies, most of them focusing on the negative effects exerted at both molecular and cellular levels, ignoring the possible benefits of free radicals. More and more people admit to having heard of the term “oxidative stress”, but few of them understand the meaning of it. We summarized and analyzed the published literature data in order to emphasize the importance and adaptation mechanisms of basal oxidative stress. This review aims to provide an overview of the mechanisms underlying the positive effects of oxidative stress, highlighting these effects, as well as the risks for the population consuming higher doses than the recommended daily intake of antioxidants. The biological dose–response curve in oxidative stress is unpredictable as reactive species are clearly responsible for cellular degradation, whereas antioxidant therapies can alleviate senescence by maintaining redox balance; nevertheless, excessive doses of the latter can modify the redox balance of the cell, leading to a negative outcome. It can be stated that the presence of oxidative status or oxidative stress is a physiological condition with well-defined roles, yet these have been insufficiently researched and explored. The involvement of reactive oxygen species in the pathophysiology of some associated diseases is well-known and the involvement of antioxidant therapies in the processes of senescence, apoptosis, autophagy, and the maintenance of cellular homeostasis cannot be denied. All data in this review support the idea that oxidative stress is an undesirable phenomenon in high and long-term concentrations, but regular exposure is consistent with the hormetic theory.

## 1. Introduction

Contemporary living standards and advances in the medical sciences promote the idea of acquiring long and healthy life by any means possible. A considerable percentage of the population is familiar with the term “oxidative stress” in association with various pathological conditions, but few people understand its beneficial role. Because of a desire to lower their oxidative status, many individuals use excessive amounts of dietary supplements containing antioxidant compounds. Therefore, the consumption of vitamins considered to be free from side effects and, at the same time, ignoring the existence of reductive stress, is one of the problems affecting the modern world. Some of the positive effects of oxidative stress and, respectively, the negative effects of reductive stress will be discussed in the following paragraphs.

In the human body there are two main types of sources for reactive oxygen species (ROS), specifically the mitochondria and nicotinamide adenine dinucleotide phosphate oxidase (NADPH oxidase/NOX). Thus, ROS generation is oxidative phosphorylation-dependent, which is a process that ensures electron flow, and which takes place in the mitochondria involving four protein complexes (I–IV) [1,2]. This process does not occur perfectly, as electron leakage can also occurs, in which electrons attach to oxygen molecules (O_2_) and thus ROS are obtained [3]. Consequently, during aerobic metabolism, the complexes that form the electron transport chain (ETC) transfer the electrons from NADH and dihydroflavin adenine dinucleotide (FADH_2_) towards mitochondrial complex IV. This complex then donates these electrons to O_2_ in order to obtain water (H_2_O). As stated previously, this process is not perfect, causing a slight percentage (<0.5%) of the electrons to react via a non-enzymatic pathway with the O_2_, forming the superoxide anion $O_{2}^{*-}$. Finally, through complexes I and III, $O_{2}^{*-}$ is released into the mitochondrial matrix and into the cytoplasm, respectively, mediated by voltage-dependent anion channels (VDACs). Concomitantly, the production rate of ROS of mitochondrial origin depends on kinetic and thermodynamic factors, on O_2_ availability, on electron transporters and, most importantly, on the membrane potential of the mitochondria [4,5]. An interesting fact is that, due to their dynamics, the mitochondria can travel across the cell interior and create areas of increased concentrations of ROS, which are made available for the transduction of cellular signaling [6].

The second source of ROS is represented by the NADPH oxidase, belonging to the NOX enzyme family. These proteins transfer the electrons from NADPH to O_2_, generating $O_{2}^{*-}$. ROS production, mediated by NOX. This transfer is regulated by flavin adenine dinucleotide (FAD), by phosphorylated proteins, and by the Ca^2+^ ion. The factors that can activate the NOX protein complex are insulin, growth factors, angiotensin, and tumor necrosis factor (TNF) [7].

Regarding the mechanisms that are supposed to limit the excess of reactive species, the human body possesses a set of proteins with an antioxidant role. Therefore, in the case of the accumulation of increased levels of $O_{2}^{*-}$, superoxid dismutase (SOD) can convert $O_{2}^{*-}$ into the peroxide H_2_O_2._ Subsequently, this set of antioxidant proteins, which can involve the peroxiredoxin (PRX)/thioredoxin (TRX) system and the glutathione peroxidase (GPX)/glutathione (GSH), system can act to reduce the previously generated H_2_O_2_ to H_2_O. This process includes the inactivation of PRX at the moment that H_2_O_2_ is reduced, the oxidation of cysteine (Cys) residues from the TRX structure, and consequently the reduction and reactivation of PRX. The oxidized, inactive TRX is then reduced by thioredoxin reductase, which has NADPH as a co-factor. The second system, which is based on glutathione, functions similarly to the one described above, except that the reduction of H_2_O_2_ is performed by GSH, which becomes oxidized glutathione (GSSG). In both systems, TRX and GSSG are reduced by NADPH, which is the result of the enzymatic activity of isocitrate dehydrogenase and malic enzymes from the pentose phosphate pathway. An important aspect is the large distribution of these antioxidant systems, granting the human organism antioxidant protection [8]. Along with these systems, nuclear factor erythroid 2-related factor (Nrf2) plays a key role, being one of the main regulators of antioxidant systems. Moreover, Nrf2 is involved in GSH regeneration by increasing glutathione reductase (GR) expression. Its molecular mechanism is discussed further in this paper [9,10].

The homeostasis of the body, tissue trophicity, and cellular signaling depend on the organic presence of ROS; therefore, the balance between oxidative stress, caused by the massive generation of reactive species, and reductive stress, caused by the excessive presence of antioxidants, must be maintained [11,12]. Cellular stress conditions, which generate sufficient ROS, promote cellular apoptosis [13] through external mitochondrial membrane permeabilization, membrane potential, and the release of cytochrome *c*. Cytochrome *c* then bonds to apoptotic protease activating factor 1 (APAF1) and forms an apoptosome, which initiates the intrinsic pathway of apoptosis via caspase 9. The intrinsic pathway involves the attachment of the cellular-death receptor Fas/CD95 and the activation of caspase-8 [14,15,16]. In certain situations, apoptosis is a beneficial process, because the abnormal cells which can no longer fulfill their function are destroyed, reducing the risk of these cells surviving and proliferating [16].

Oxidative metabolism of the cell generates reactive species, with very high energy, that can interact with susceptible endogenous molecules, such as proteins, unsaturated fatty acids, and DNA [17,18,19,20]. Overall, oxidative stress is described as a redox ratio between antioxidants and oxidizing agents, favoring the latter, which leads to the assumption that reactive oxygen and nitrogen species (RONS) [21] present only an intrinsic negative effect [22,23]. This definition is not completely accurate as a moderate level of ROS and/or reactive nitrogen species (RNS) is needed to maintain cellular homeostasis [24,25,26,27,28] due to their involvement in immune mechanisms, angiogenesis, adaptation, and muscle remodeling [29,30,31,32].

The presence of ROS in small or moderate doses is considered to be beneficial for physiological functions involved in the progression of the cellular cycle, such as differentiation, the development of cells, and cellular apoptosis. Reactive species are involved in different phases of cellular signaling, often via the reversible oxidation of thiol groups (-SH) on the proteins, which will be described in the following sections. One of these phases is the activation of some transcription factors, such as the phosphoinositide 3-kinase/protein kinase B system (PI3K/Akt system), mitogen-activated protein kinase (MAPK), nuclear factor erythroid 1- and 2-related factors (Nrf1, Nrf2), Kelch-like ECH-associated protein 1 (Keap-1), and nuclear factor kappa B (NF-kB). Moreover, the presence of ROS is essential for the activity of the immune system and for maintaining redox balance through the activation of antioxidant systems. The modification of this balance in favor of ROS production is often linked to pathological states, diabetes mellitus, atherosclerosis, cancer, or neurodegenerative diseases (such as Alzheimer’s and Parkinson’s). Despite previous beliefs, ROS is associated with these conditions, rather than being a direct cause of them. On the other hand, the inadequate use of supplements based on antioxidants can cause a paradoxical state of “antioxidant stress”. The efficiency of the antioxidant and homeostatic systems is dependent on ROS exposure, since the expression of the proteins involved in these processes is increased by the presence of oxidative stress. The improvement of antioxidant activity is therefore due to the presence of an oxidative state, given that some health aspects are linked to pro-oxidant factors such as physical exercise.

Based on the above considerations, it can be stated that the presence of an oxidative state is required for the proper functioning of the body, having well-defined roles, but to date it has been insufficiently researched and explored.

## 2. Influence of Oxidative Stress on Physical Activity

Physical activity, in addition to improving health status and preventing the development of pathological conditions, is responsible for producing reactive species. However, the human body is able to reduce free radicals through several mechanisms and they do not represent a threat under physiological conditions [33]. The correlation between the type of physical activity and oxidative stress, and its beneficial effects on health and redox balance are still subjects of controversy [34].

Since the energetic needs and oxygen consumption levels are different, the generation of ROS during physical activity depends on many factors, such as the intensity type of physical effort and its duration. In cases of physical activity of reduced intensity and duration, the body’s antioxidant systems are sufficient to maintain the redox balance. An increase in duration and intensity becomes challenging, and in this case oxidative lesions can be observed. However, chronic exposure to oxidative stress due to regular physical exercise does not lead to cellular lesions, but it is rather—in accordance with the theory of hormesis—an adaptive mechanism [35]. The type of physical exercise plays a crucial role regarding oxidative stress; illustrative examples include the cycling exercise in which the activity of the antioxidant systems is increased [36,37]. In contrast, for the same intensity of some anaerobic exercises (sprints, intermittent running, isometric exercise, jumps), oxidative injuries can be observed. [38]. The latter is due to the activation of xanthin-oxidase (XO), which produces ROS during ischemia reperfusion. XO reduces O_2_ to $O_{2}^{*-}$ and H_2_O_2_, which is then reduced to hydroxyl (${\mathrm{HO}}^{*}$), which is considered to be responsible for these cellular lesions [39]. The underlying mechanisms of the increase in ROS could be moments of ischemia-reperfusion, as well as mechanical stress, muscular injury, and the migration of the inflammatory cells to the affected area. Following the same model, endurance physical exercise enhances antioxidant enzymatic activity, indicating that the type and intensity of physical activity are predictors of oxidative stress generation [40]. In summary, the abovementioned factors can either have negative effects or positive effects or both, depending also on the individual basal levels of oxidative stress. An important factor to be mentioned, which is involved in the adaptive mechanism and antioxidant protection, is gender, but this is outside of the scope of this paper and will not be further discussed.

Even though the role of oxidative stress in effort-induced adaptation remains to be elucidated, one thing is certain: an optimal yet undefined level of ROS is mandatory for the proper function of the adaptive mechanisms. Similarly, to aerobic activity, it is unclear if ROS generated during anaerobic exercises represent an adaptive/necessary event or are harmful, and studies in this area are rather scarce [41].

Striated muscular fibers react differently in the presence of ROS/ RNS, depending on the degree of stress adaptation. As such, for people who perform physical activity on a regular basis, an increase in the intensity of physical exercise is correlated with better glucose metabolism, an increase in the number of mitochondria, and muscular hypertrophy [42,43,44]. On the contrary, for physically inactive people, the trophicity of the muscular tissue is affected [45,46,47,48,49,50].

Many studies suggest that moderate-intensity exercise is responsible for generating a beneficial level of reactive species [28,51,52,53], as physical activity is associated with health and slowing down the process of aging [54,55,56] by increasing the expression of antioxidant enzymes [51,57].

The ability of the human body to adapt to oxidative stress produced during endurance exercise is a result of an increased mitochondrial volume and a decreased inflammatory response [23,58,59,60]. As the free radicals $O_{2}^{*-}$ and ${\mathrm{HO}}^{*}$ have a short half-life and cannot be quantified, oxidative damage is determined indirectly through biomarkers such as oxidized low-density lipoprotein (oxLDL) [61], malondialdehyde (MDA) [62,63], the reduced and oxidized glutathione ratio (GSH/GSSG) [64], F2-isoprostane [65,66], carbonylated proteins [67,68], or 8-hydroxy-2′-deoxyguanosine (8-OHdG) [69,70], which are lower in physically active individuals than in sedentary and inactive ones [32]. Sedentarism is a risk factor for muscular atrophy, a process that paradoxically produces ROS and decreases the ability of the cells to trap free radicals [71]. Transitory oxidative status is required during physical effort to initiate the adaptive mechanisms of skeletal muscles. Based on these hypotheses, the hormesis theory was formulated [56,72]. This theory claims that continuous exposure to low level of stress factors improves the ability of the cells to respond to higher levels of stress [73] due to the phenomenon of the up regulation of endogenous antioxidant systems. The exact pathways through which reactive species that are produced during physical effort can induce adaptive mechanisms are not completely known, but it seems that enzymatic systems [74,75] that increase the expression of manganese-dependent superoxide dismutase (MnSOD) [51], catalase (CAT) [52], and glutathione peroxidase (GPx) are activated [76,77,78].

NF-kB is activated in the presence of pro-inflammatory cytokines, TNF-α, interleukins 1 and 6 (IL-1, IL-6) [79,80], ionizing radiation [81,82], and other stressors, with the signaling molecule being H_2_O_2_ [30] acting through the dissociation of kappa B inhibitory factor (I-kB) [83,84] and translocation into the nucleus [76]. Moreover, in order to improve the vascularization of striated muscles via NF-kB and hypoxia-inducible factor 1 (HIF-1)—which is associated with peroxisome proliferator-activated receptor gamma coactivator 1-alpha (PGC-1α) [85,86,87] and ROS and is especially, RNS-dependent through nitric oxide (NO)—angiogenesis is favored. High concentrations of H_2_O_2_, together with $O_{2}^{*-}$, are considered risk factors due to their pro-inflammatory effect, resulting in a thickening of vascular walls and the occurrence of associated vascular diseases. Vascular endothelial growth factor (VEGF) activates ROS-generating NADPH oxidase/NOX [88] and triggers cell proliferation and migration [89], subsequent to the stimulation of endothelial cells by the tyrosine kinase receptor (RTK) VEGF 2 [90]. Several signaling pathways, such as MAPK, Akt, and endothelial nitric oxide synthase (eNOS), are activated in these chain reactions that are essential for the neovascularization process [91]. Regarding these considerations, regular exercise of moderate intensity respects the theory of hormesis due to the ability of the body to adapt to stronger oxidative stressors.

**Figure 1:**
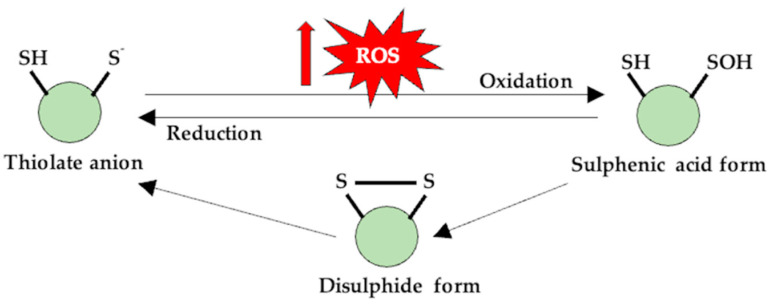
Thiol (-SH) groups are susceptible to redox reactions, and at physiological pH values, they are found in an anionic form. The oxidation of this form generates a sulfenic derivative, which can reverse to its initial form through the disulfidic form, and, in this case, the protein can change its functionality.

The elevated levels of ROS that occur in both performance athletes and individuals who practice sports for recreational purposes cause variable cellular adaptive mechanisms that depend on several factors such as the origin and the concentration of reactive species, on the exposure time to ROS and, especially, on the particular/individual oxidative status. Thus, RONS act as messengers, signaling physiological regulatory functions, through mechanisms involving post-translational redox changes of -SH groups on Cys residues [92]. Redox signaling is based on the structural modification of compounds such as Cys, although other protein molecules, as well as other signaling pathways that use various kinases and/or phosphatases producing phosphorylation and/or dephosphorylation reactions, are also involved [93,94,95], as shown in Figure 1. Therefore, by modulating the Cys residues, ROS can activate or inactivate other proteins. For RTKs, ROS generation is necessary for the downstream signaling. Phosphorylation of tyrosine (Tyr) residues requires the inactivation of tyrosine-phosphatase (PTPs). These enzymes are dependent on the Cys residues which are located in their active center, and the oxidation of the -SH groups inhibit enzymatic activity [96]. Moreover, ROS can also modulate the extracellular part of the RTKs, hence activating it in the same way that they inactivate the PTPs. [97]. Contrary to previous hypotheses, the main source of ROS is not mitochondria but rather enzymatic sources such as NOX(s), phospholipase A2 (PLA2), and/or XO [50,98,99]. ROS-based signaling pathways are involved in various acute and chronic skeletal muscle responses to exercise, from increased insulin sensitivity [100] to the modulation of endogenous enzymes with antioxidant activity [101], mitochondrial biogenesis [102], and the modification of muscle contraction and adaptation [103]. Regarding the generation of RNS during muscle contraction, NO is produced by neuronal nitric oxide synthase (nNOS). Post-translational reversible changes, such as S-nitrosylation, S-glutathionylation, sulfenylation, or the formation of disulfide bonds by Cys-reactive thiol groups, are in fact redox changes—the response of the cells to abnormal ROS and RNS levels [104,105,106], as shown in Figure 1.

For example, through the S-nitrosylation process, the activity of proteins involved in muscle contraction—ryanodynamic receptors (RyR1), myosin, cAMP response element-binding protein (CREB), proteins specific to insulin signaling—are altered [107,108,109,110], but at the same time, these reversible redox changes in the -SH groups of Cys also have a protective role for proteins as they make them less susceptible to oxidative processes [104].

For optimal muscle contraction, a certain level of reactive species is required, because depending on its level, the contraction of skeletal muscle can be improved by increasing the sensitivity of muscle fibers to the Ca^2+^ ion, whereas post-effort, when the ROS level is high in the muscle, the contraction force and sensitivity to Ca^2+^ is decreased [111]. For this reason, the administration of dietary supplements with antioxidant compounds may not be beneficial as it might decrease the effort capacity [111,112,113]. In a study performed by Reid et al. the hypothesis of a “redox break” was introduced, stating that fatigue and decreased muscle contraction is due to the generation of high levels of ROS, and it is actually a negative feedback mechanism through which the damage caused by ROS to the skeletal muscle is minimized [114,115]. In addition to altering the proteins involved in regulating the Ca^2+^ concentration or the sensitivity of the muscle fiber to this ion, they propose the hypothesis that ROS influences Na^+^/K^+^ pump activity and thus reduces muscle contraction force in the case of endurance-based physical effort [116]. Physical effort causes depletion of K^+^ ion sand a massive increase in the intracellular concentration of Na^+^ ions, which affects the membrane excitability, and therefore the force of muscle contractions is diminished. This idea is supported by the experimental data showing that the administration of an antioxidant, N-acetylcysteine (NAC), reduces muscle fatigue that occurs during exercise by regulating the intracellular concentration of K^+^ ions [117].

There are various communication pathways that use redox signaling and the most important ones that also have an impact on physical effort are NF-kB, MAPK, and PGC-1α, and their activity is influenced by the concentration of H_2_O_2_ [118]. Another important aspect to mention is that these communication pathways are interdependent and intersected, through a process known as “crosstalk” [119]. Studies also present data confirming the influence of PGC-1α on mitochondrial function and dynamics (fusion, fission) on muscle fiber differentiation and on the expression of antioxidant enzymes, which are present in oxygen-consuming tissues (brain, heart, skeletal muscle) [120,121].

The mitochondrion cellular organelle is of vital importance. It exhibits bioenergetic and functional roles in maintaining cell viability, throughout the many biochemical processes that are under its control (the Krebs cycle, β-oxidase, oxidative phosphorylation, OXPHOS). Concomitantly, the mitochondrion is a dynamic organelle as it can switch its morphology depending on intra- and extracellular conditions in order to preserve cellular homeostasis. Thus, the presence of a stressor, for example, food deprivation, activates the mitochondrial fusion process, controlled by optical atrophy 1 (Opa1) and mitofusin 1/2 (Mfn1/2), to grow and enhance the OXPHOS rhythm. It is believed that mitochondrial fusion increases activity and energy production, whereas fission precedes mitophagy.

On the opposite side, in the case of nutrient excess, fragmentation of mitochondria takes place, also known as fission [122,123], controlled by a pair of proteins, dynamin-related protein 1 (Drp-1) and mitochondrial fission 1 protein (Fis1) [98,124,125].

**Figure 2:**
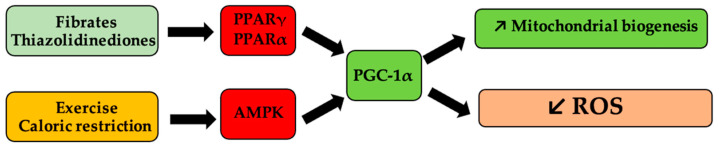
Schematic representation of mitochondrial biogenesis pathways of influence and of reactive oxygen species (ROS). Antihyperlipidemic medication, such as fibrates and antihyperglycemic medication, such as thiazolidinedione derivatives, activate peroxisome proliferator-activated receptor gamma coactivator 1-alpha (PGC-1α) via peroxisome proliferator-activated receptor γ and α (PPARα, PPARγ), respectively, and determine the onset of mitogenesis and the decrease in ROS levels. Similarly, caloric restriction or physical effort, via activation of the same receptors, following the activation of 5′ protein kinase by the AMP (AMPK) pathway, lead to the same processes.

Mitochondrial homeostasis and biogenesis can be pharmacologically modulated by agonists (thiazolidinediones, fibrates) of peroxisome proliferator-activated receptor γ, and α (PPARγ, PPARα), respectively [126,127], as shown in Figure 2.

During physical activity, PGC-1α expression is elevated as a result of the increase in the concentration of Ca^2+^ and activation of Ca^2+^/calmodulin-dependent protein kinase II (CaMKII), CREB, and myocyte enhancer factor-2 (Mef2) [128,129,130,131,132]. Meanwhile, under the action of PGC-1α, the number of mitochondria is high within striated muscle, and as such, energetic resources are more efficiently used [133,134,135,136,137]. Numerous animal and human studies demonstrate that moderate-intensity physical effort increases the values of mitochondrial biogenesis markers and the expression of antioxidant systems, demonstrating the appearance of the adaptative response [138,139]. More and more data suggest that physical effort has an overall antioxidant role, depending on the type of exercise [140], as well as age and sex, which is a cause of controversy in the hormesis theory. Thus, endurance physical effort is a massive consumer of energy, which increases the level of adenosine monophosphate (AMP) with the activation of the 5′ protein kinase activated by AMP (AMPK) [141], MAPK, CAMKII, and Mef2 [124], which triggers the activation processes of PGC-1α, which is necessary for the adaptative mechanism of muscle tissue and the transition of glycolytic fibers to oxidative fibers [141,142,143,144].

**Figure 3:**
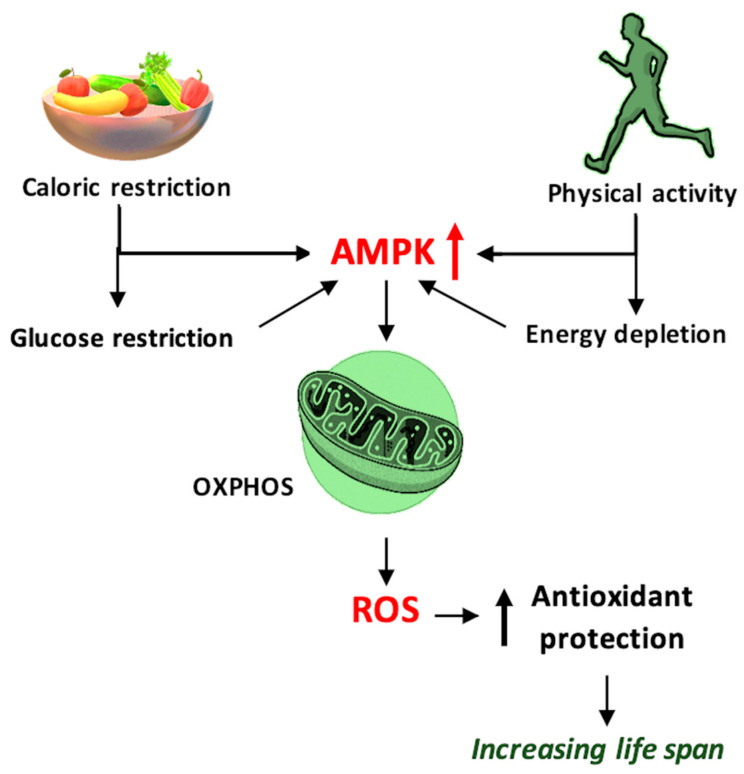
Schematic representation of the mechanism by which caloric restriction and physical activity prolong the lifespan and improve health, using 5′ adenosine monophosphate-activated protein kinase (AMPK) and reactive oxygen species (ROS) as mediators, and ROS generated through oxidative phosphorylation (OXPHOS)—schematic mechanism.

At the same time, the skeletal muscle is a consumer of adenosine triphosphate (ATP), so it creates an energy deficit with the generation of high concentrations of AMP, which activates AMPK, another determining factor in increasing PGC-1α expression [136,137], as shown in Figure 3. On the other hand, for the proper functioning of the muscles during physical effort and in order achieve optimal muscle contraction, the presence of NO, produced during physical movement, is necessary. NO increases blood flow and improves the antioxidant status of skeletal muscle and is synthesized via inducible NO synthase (iNOS), an enzyme of which the expression is induced by physical movement via NF-kB [131]. Although the NF-kB pathway is important for maintaining normal oxidative status, chronic activation may have adverse effects such as amplifying the synthesis of pro-inflammatory cytokines that generate ROS, inhibit PGC-1α activity, and promote apoptotic processes [138]. All these processes are not universal, but are influenced by certain variable factors, such as age, sex, eating habits, and even the physiological condition of an individual.

**Figure 4:**
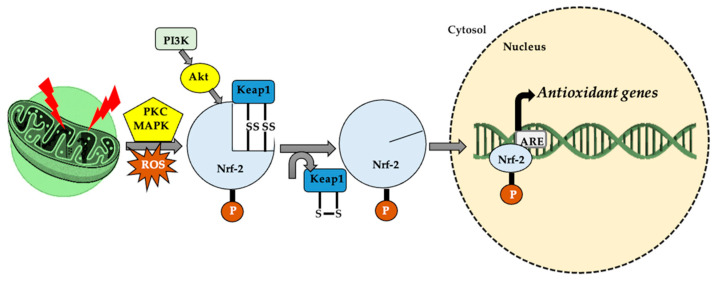
In cellular homeostasis, nuclear factor erythroid 2-related factor (Nrf-2) stabilizes by attaching to Keap-1 in the cytoplasm. The appearance of a stressor/reactive oxygen species (ROS) generator, under the action of protein kinase C (PKC), mitogen-activated protein kinase (MAPK) and phosphoinositide 3-kinase–protein kinase B (PI3K–Akt system), the thiol (-SH) groups belonging to Kelch-like ECH-associated protein 1 (Keap-1) are oxidized (S-S cross links). This leads to Nrf-2 dissociation and translocation in the nucleus, where it attaches to a specific sequence, antioxidant responsive element (ARE), thus increasing cellular antioxidant activity.

In patients with type 2 diabetes, physical effort improves glycemic status [139,140], one of the mechanisms involving oxidative stress, which is correlated with the expression of the glucose transporter type 4 (GLUT4) [141,142] and the involvement of proteins such as AMPK, PGC-1α [143], and MAPK [140,142]. The activation of PGC-1α as a result of aerobic effort [144,145] and an increased AMP/ATP ratio [146] decreases oxidative stress and inflammation and increases mitochondrial biogenesis [147,148] in response to the interaction with Nrf-1 [35] and Nrf-2 [88,147] and promotes muscle adaptation mechanisms following AMPK activation [31,148,149]. AMPK is an energy sensor with an important impact on metabolic status and on the mRNA expression of PGC-1α, requiring the presence of ROS. This fact was demonstrated using antioxidant compounds that suppressed the adaptive mechanisms and the formation of PGC-1α [132,150,151]. Regarding Nrf-2, in oxidative stress conditions, the oxidation of -SH groups from Kelch-like ECH-associated protein 1 (Keap-1) allows Nrf-2 to translocate in the nucleus [115,152], subsequent to the separation of Keap-1 [153]. In response, an increase in the expression of the antioxidant responsive element gene (ARE) occurs [154,155,156], which controls the level of glutathione reductase (GR) and glutathione synthase (GSS) [157], acting as a mediator between oxidative and reductive stress [158], as shown in Figure 4.

Thus, ROS are sources of oxidative stress, but they are necessary in small amounts in order to maintain physiological functions, as well as for muscle contraction. However, an excessive increase in ROS during intense physical activity can have detrimental effects on performance.

## 3. Impact of Oxidative Stress on Aging

It is known and generally accepted that mitochondria are responsible for generating reactive species and their excess causes oxidative lesions and cellular aging. However, as previously mentioned throughout this paper, ROS also play a role in cellular signaling. Thus, the existence of two types of ROS can be discussed: species with low reactivity ($O_{2}^{*-}$, H_2_O_2_) and species with high reactivity (${\mathrm{HO}}^{*}$, peroxynitrite, ${\mathrm{ONOO}}^{-}$). Although the species with low reactivity act as cellular messengers, the ones with high reactivity contribute to oxidative injuries, to the diminishing of cognitive capacities, brain aging, and finally to the onset of neurodegenerative diseases [6].

Studies carried out on *Caenorhabditis elegans* presented interesting data regarding aging processes. They indicated that glucose deprivation increased OXPHOS and mitochondrial ROS production (mtROS) but diminished cellular aging and increased life expectancy. This phenomenon, in which the adaptation of mitochondria takes place as a consequence of exposure to increased levels of ROS and the activation of antiaging mechanisms occurs, is referred to as mitohormesis [159,160]. For this phenomenon to take place, the properties of the mitochondria have to be intact. One can infer that with increased O_2_ availability, ROS production increases simultaneously. In fact, studies indicate that hypoxic states are correlated with increased or even accelerated production of ROS and also with increased aging processes. Thus, during hypoxia, inhibition of mitochondrial complex III generates ROS and alters the cellular response in this particular state, by impeding HIF-1α stabilization [161]. Another relevant example is the linkage between mtROS and uncoupling protein 2 (UCP2). During periods of low nutrient intake, mtROS is generated via the β-oxidation process. This increases the activity of UPC2, which preserves a low level of ROS, but maintains the processes responsible for the generation of energy. In cases where this redox balance, maintained with the active participation of mitochondria, is altered, the activation of UCP2 and the production of energy are impeded. This can partly explain the ineffectiveness of antioxidant therapies in neurodegenerative diseases. Based on this fact, treatments dedicated to these particular diseases should aim to reach or maintain redox homeostasis, rather than aiming at the reduction of the total levels of ROS [162].

In the opposite situation, the altered and non-functional mitochondria are eliminated via mitophagy and the proteasome system [163]. On this note, studies state that an increase in the activity and effectiveness of mitophagy and the proteasome system can increase life expectancy and slow down the aging rate, at least in some model organisms (rodents, flies, and worms) [164,165,166]. However, the notion that the mere presence of functional mitochondria would prevent aging or the manifestation of already-present neurodegenerative diseases remains unclear.

Diseases with neurodegenerative components are characterized by affected functions as well as progressive destruction of neuronal cells. The modification of redox homeostasis is a specific attribute of neurodegenerative conditions and despite the fact that in some experimental models of Alzheimer’s, Parkinson’s, or other neurological conditions antioxidant agents demonstrated beneficial effects, in clinical studies, this neuroprotective effect could not be demonstrated [167,168].

From a morphological perspective, the destruction of neuronal mass is linked to protein misfolding and the accumulation of misfolded protein aggregates. An interesting fact is that some neuronal populations are resistant, whereas some areas are sensitive to oxidative stress, a phenomenon termed selective vulnerability. Finally, the association between protein aggregates and oxidative stress triggers a chain reaction, which leads to neuronal death [169].

In the case of the dysfunction of the proteasome system, which is responsible for the disposal of the altered proteins, and which is therefore indispensable for maintaining cellular homeostasis and neuronal survival, the protein aggregates can trigger the generation of ROS. This phenomenon is often encountered in diseases with an oxidative component, especially the neurodegenerative ones. It is known that the brain does not possess a remarkable antioxidant capacity, which makes it susceptible to oxidative damage, particularly during aging, when the antioxidant systems are less active and favor the accumulation of reactive species and the progression of diseases such as Alzheimer’s and Parkinson’s [170,171,172].

Alzheimer’s disease is a widespread neurodegenerative condition, involving the progressive loss of memory, cognitive capacities, and personality disorders as the most important symptoms. Morphological particularities in Alzheimer’s are represented by amyloid plaques, dystrophic neurites, cerebral amyloid angiopathy, reactive astrogliosis, as well as microglial activation [173]. The absence of the protein Nrf-2 from the nucleus of the hippocampal neurons is implicated in this disease, which can be confirmed through biochemical analysis of the patient’s brain. Concomitantly, it is likely that in these patients, the response of Nrf-2 to ROS and the mitochondrial dynamics are affected, which further leads to the progression of molecular mass loss [174]. The accumulation of β-amyloid and Tau proteins is age-dependent—it increases as one person gets older, and this accumulation disrupts Ca^2+^ ion homeostasis and the mitochondrial membrane potential is lost. Furthermore, the internal membrane develops pores and ATP synthesis decreases, but the ROS level increases as a result of electron drain.

Regarding Parkinson’s disease, which is the second most widespread neurodegenerative condition after Alzheimer’s disease [175], it mostly constitutes motor symptoms (tremor at rest, bradykinesia, muscular rigidity) and non-motor symptoms (constipation, depression, personality disorder). The symptomatology is the result of the degeneration of dopaminergic neurons in the substantia nigra [176,177]. In this case, the presence of ROS leads to Nrf-2 activation, which further disrupts α-synuclein (α-Syn) metabolism, which would thus prevent the loss of neuronal dopaminergic mass. However, in patients diagnosed with Parkinson’s disease, a deficit of the Nrf-2 protein is observed, and this deficit causes neuroinflammation and favors the progression of the disease [174]. In both neurodegenerative diseases, age is one of the most important determining factors of the onset and/or the progression of the disease.

It is generally accepted that oxidative stress is involved in various diseases, such as neurodegenerative diseases (Alzheimer’s, Parkinson’s), but it is possible that ROS are not actually the trigger for the disease but rather the cause of the exacerbation of symptoms within these neurological disorders [178,179]. Therefore, various studies have been performed to investigate the influence of oxidative stress and physical effort on central nervous system conditions. The data presented in the literature demonstrate an inversely proportional relationship between the incidence of neurodegenerative diseases and physical activity, and a slowdown of their progression in the case of physical activity [135,180,181,182,183].

Moreover, as neuronal metabolism increases, there is a consecutive increase in oxygen consumption and ROS production, triggering a positive feedback effect on antioxidant mechanisms and neurotrophin synthesis, which improves cognitive functions and offers protection against neuronal degradation [184]. Brain-derived neurotrophic factor (BDNF) is required for neurogenesis and neuronal plasticity [159,185], and its release is dependent on oxidative status [88] and caloric restriction (CR) [186,187]. Evidence to support these hypotheses was obtained through a study on Alzheimer’s disease [188], in which a decrease in the accumulation of β-amyloid [189] was observed, possibly due to the proteolysis and autophagy of denatured proteins [190], and the overexpression of neuroglobin as a protective factor of the brain from the neurotoxic action of β-amyloid [188]. The data presented in the literature suggest that the moderate level of reactive species formed during exercise favors the expression of 8-oxoG DNA glycosylase [190], an enzyme involved in the repair of nucleic acid chains due to the accumulation of 8-OHdG [52,184]. Preventing the accumulation of degraded DNA presents beneficial effects on senescence and other associated degenerative diseases [191]. A study conducted by Radák et al. confirmed the beneficial effect of moderate physical exertion on neurodegenerative diseases, mediated by proteasome activity and the degradation of altered proteins [192].

ROS are also involved in the formation and storage of memories via improving synaptic plasticity [192], suggesting the modulation of cognitive capacity, which entails the activation of hormetic processes at the neuronal level. The generation of free radicals associated with the influx of Ca^2+^ ions activate transcription factors CREB and NF-kB, causing a release of cytoprotective proteins [193]. Thus, it can be concluded that exposure of the brain to moderate levels of reactive species improves endurance and slows down the aging process of neuronal tissue [194]. Altogether, these data show a neurohormetic effect that can suppress the progression of neurodegenerative diseases [195].

This is a paradox—exercise-induced oxidative stress, by activating and improving adaptive mechanisms, is responsible for improving overall physiological function, reducing the prevalence of various diseases, increasing the quality of life, and, consequently, inducing the adaptability of the body to oxidative stress [196]. Thus, increasing the body’s capacity to trap and neutralize ROS can provide better protection and resistance during training and can also intervene in reducing the aging process. A study performed by Bouzid et al. supports the hypothesis that the aging process alters antioxidant activity regardless of the physical activity level, thus affecting both sedentary and active people. However, regular physical activity improves antioxidant capacity in the young population to a greater extent than in the elderly, suggesting that the beneficial effects of regular physical activity could be affected by the aging process. In addition, the study data support the idea that regular physical activity reduces the aging process [197].

Overall, physical activity must be performed throughout the lifetime in order to maintain the benefits of adaptive mechanisms following exercise-induced oxidative stress [197]. These data are supported by other studies concluding that with age, muscle mass decreases, a process known as sarcopenia. There are several factors involved in the development of sarcopenia, such as neuro-endocrine factors, nutritional factors, decreased physical activity, and especially oxidative stress caused by mitochondrial dysfunction [198,199,200]. The mitochondrial theory of sarcopenia claims that muscle weakness occurs due to decreased oxidative capacity of mitochondria and thus a reduced amount of generated ATP. This is due to the decreased ability to replace altered mitochondrial proteins involved in the ETC with age [201,202]. In addition, in senescent muscles, NF-kB is constitutively activated, which leads to increased production of pro-inflammatory cytokines and ROS. At the same time, the forkhead family of transcription factors (FoxO) is activated through ROS, which decreases the expression of PGC-1α and favors the degradation of the neuromuscular junction and mitochondrial trophicity [203].

Unlike the past decades, in which ROS were considered the main cause of the aging process, it has been confirmed that small amounts of free radicals prevent senescence and improve the lifespan and the quality of life. Another associated theory is related to CR, demonstrated by the ketogenic diet [54,194,204,205], as it improves antioxidant activity and reduces the amount of ROS to a level that slows down senescence. The exact mechanism through which CR reduces oxidative stress is not completely known and available data are ambiguous. A potential mechanism could involve increased degradation of the modified molecular structures with a consecutive increase in the synthesis of new molecules to replace them, as well as a simultaneous decrease in the rate of production of free radicals by mitochondria [123]. With age, carbonylated proteins tend to accumulate as their degradation mechanisms decrease, but in physically active people due to ROS produced during exertion, the expression of the proteasome complex [184,206], responsible for protein degradation, is potentiated [207].

Most frequently, the aging process is associated with the loss of tissue, the underlying theory being that of oxidative stress coupled with lesions caused by ROS. Thus, sustained physical activity is related to a better neutralization of ROS, which leads to protection against oxidative injuries and the prevention of age-related conditions.

## 4. A Link between Oxidative Stress and Immune Response

In a general sense, a compound with antioxidant properties is considered to be able to neutralize the RONS species either directly, indirectly, or by inhibiting their subsequent generation [208]. As a result, antioxidants can be classified according to their mechanism of action [209,210] into:

- enzymes (SOD, CAT, GPx), proteins (ceruloplasmin, ferritin), and minerals (zinc, copper, selenium) considered to be agents that prevent the formation of new free radicals;
- agents considered to be scavengers act by inhibiting the initiation and propagation of peroxidation reactions (vitamin C, vitamin E, β-carotenes, glutathione, flavonoids);
- enzymes involved in the reconsolidation of affected cell membranes (lipases, proteases, transferases, DNA repair enzymes).

Considering that the theory of hormesis is generally accepted and that RONS are necessary for cellular homeostasis, muscle regeneration, slowing down the process of aging, protein degradation, and cellular signaling, it is still not possible to determine if a reductive status of the body is beneficial or not, as it generates reductive stress [211] that causes cellular metabolism disorders and cytotoxicity [212]. Nonetheless, the literature emphasizes the involvement of mitochondrial oxidative metabolism in stem cell differentiation, such that complete differentiation is dependent on the presence of ROS [213,214,215,216,217,218]. This information does not exclude the fact that ROS or increased levels of ROS are not harmful, but there are circumstances and types of cells (adipocytes, muscles) of which the differentiation and renewal depend on the organic presence of these species [219,220]. In the meantime, ROS influence the successive phases that occur in the cellular cycle, and the administration of the compounds that donate -SH groups (NAC) apparently cause cycle arrest in the G_1_ phase, whereas in the absence of these compounds, the S phase is prolonged and the G_1_ phase is shortened [221,222]. Studies demonstrate that antioxidants have a wide range of beneficial effects, with most of these studies conducted in conditions of induced oxidative stress and with only a few studies questioning what happens under normal physiological conditions [223,224].

Therefore, in the absence of an oxidative status, the self-renewal capacity of cells and autophagy processes are disrupted, with the accumulation of chromosomal defects and abnormalities and the inability to repair DNA and induce apoptosis [225,226]. Highly active antioxidant systems induce reductive stress, influencing [130] cell growth and mitochondrial functions and are incriminated in the progression of pathological conditions such as Alzheimer’s disease [227] because of an impaired protein folding process [12], or cardiomyopathy, cancer, or metabolic syndrome [86,228,229,230]. Some examples of the negative influence of the most frequently used antioxidant substances are illustrated in Table 1.

It was initially considered that reductive stress is preceded by oxidative stress, but the relation between the two concepts is reciprocal [253]. High doses of antioxidants did not provide conclusive data [193,254] regarding their influence on lifespan [122,255] and, on the contrary, created a pro-oxidant status with the alteration of the redox balance. The accumulation of reduced forms of coenzymes (NADH and NADPH) and reduced glutathione (GSH) [256] can cause cellular dysfunction. Furthermore, mitochondria generate ROS in response to the accumulation of reductive stress. One example is vitamin C (an antioxidant and reducing agent at the same time), which reduces Fe^3+^ to Fe^2+^, and thus has the ability to re-enter the Fenton reaction, generating $O_{2}^{*-}$, followed by its transformation into HO* radicals [193].

In addition to vitamin C, other compounds, including resveratrol and lipoic acid (LA), can be mentioned, but the phenomenon is not limited to these examples. Thus, in the case of resveratrol, a series of beneficial effects correlated with its antioxidant properties was observed: cardioprotection, neuroprotection, anti-inflammatory, and antimicrobial activities, and it also mimics the effects of CR, which are correlated with lifespan [257]. Moreover, some studies demonstrated effects on ovarian and testicular activities, prolonging their viability as a result of a potential antiaging effect [258]. On the contrary, resveratrol can also present negative effects [259], associated with its pro-oxidant behavior. Depending on the enzymes involved, the compound can be oxidized and/or auto-oxidized, resulting in semiquinones and 4′-phenoxyl radicals, responsible for ROS generation [260], which can alter the normal functioning of lipid membranes and/or genetic material [261]. It is also important to mention that excessive doses of resveratrol can present other adverse effects which are not correlated with oxidative stress, such as impeding cellular growth and the activation of apoptosis in normal cells, atherogenesis, and phytoestrogen-like behavior [262,263,264].

LA, also known as α-lipoic acid and/or thioctic acid, displays biochemical roles and antioxidant properties. LA is reduced to dihydrolipoic acid (DHLA) by GR and TRX in the presence of NADPH. Both molecules, LA and DHLA, exhibit their antioxidant properties in hydrophilic as well as in lipophilic media because of their amphipathic character [265]. The racemic mixture of LA is used in therapy, especially in diabetic neuropathy. Its antioxidant properties are only evident in the interaction with other antioxidant systems. For example, the capacity of reducing GSSG to GSH is based on the initial reduction of LA to DHLA, which further reduces cystine to Cys, and the latter is used for GSH synthesis [266]. On a mitochondrial level, along with other antioxidants (GSH, vitamin C, and vitamin E), LA increases the activity of the enzymes involved in the Krebs cycle [267]. Furthermore, in a study conducted by Marangon et al., supplementation with LA resulted in diminishing urinary levels of F2-isoprostan and oxLDL [268]. A potential antiaging effect based on the protection of cardiac cells in aged rodents was also suggested [269].

However, its pro-oxidant properties, demonstrated in in vitro and in vivo studies, cannot be excluded. In vitro, DHLA forms chelates with both ferric and ferrous ions, which can extract the iron from ferritin and reduce it to Fe^2+^, at the same time increasing the possibility of oxidative processes via the Fenton reaction. In another study conducted by Çatakay et al., it was observed that the administration of LA in aged rats resulted in higher levels of carbonylated proteins compared to the control group [270].

The idea that substances/compounds with antioxidant activity are harmless is imprinted in popular belief and this conception is also supported by the media. However, there are questions as to how antioxidant compounds intervene in slowing down the aging process and the appearance of various diseases. The ability of antioxidants to prevent the accumulation of oxidizing molecules, thus helping to prevent aging, cancer, and even infections, has been also questioned.

The adaptation capacity of the immune system is essential for antimicrobial defense, as well as for the immunological memory. In this sense, the presence of mitochondria is mandatory, as it facilitates the aforementioned antimicrobial defense through ROS generation. At the same time, mitochondria are necessary for T-cell activation, which generates ROS and activates the nuclear factor of activated T cells (NFAT), which further enhances IL-2 synthesis [271,272]. This statement is supported by research studies that demonstrated that T-cells, for which antioxidant agents were made available, showed reduced levels of IL-2 and a reduction in the degree of proliferation was observed, sustaining an immunosuppressive effect resulting from the exposure to antioxidant agents. The same effects could also be noted in the case of T-cells that lacked mitochondrial complex III, supporting the importance of ROS in the immune response [273].

**Figure 5:**
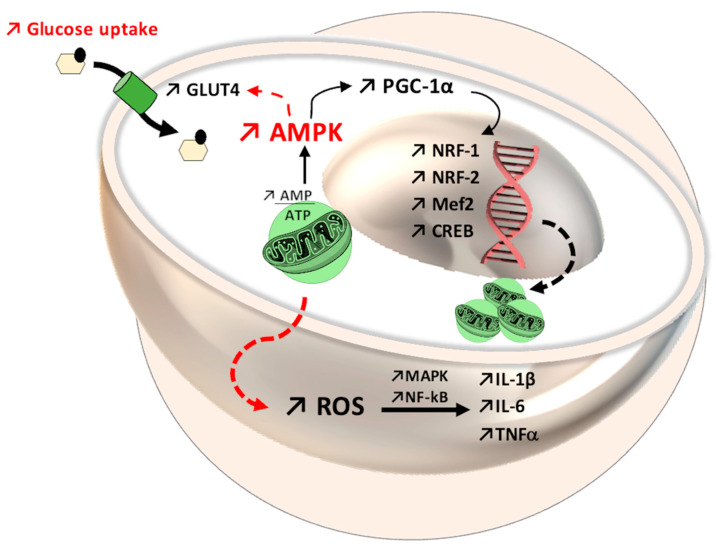
Mitochondrial dynamics through the 5′ adenosine monophosphate-activated protein kinase-peroxisome proliferator-activated receptor gamma coactivator 1-alpha (AMPK-PGC-1α) system, with increased expression of nuclear factor erythroid 1-related factor (Nrf-1), nuclear factor erythroid 1-related factor (Nrf-2), myocyte enhancer factor-2 (Mef2), and cAMP response element-binding protein (CREB). At the same time, in the AMPK pathway, an improvement in glycemic status can be observed, through translocation of glucose transporter GLUT4 in the cellular membrane. Regarding the immune system, reactive oxygen species (ROS) enhance the activity of mitogen-activated protein kinase (MAPK) and nuclear Factor kappa B (NF-kB), which further favors interleukin and cytokine secretion (IL-1β, IL-6, tumor necrosis factor, TNF).

Macrophages and the neutrophils constrain the ability to phagocytize foreign compounds/microorganisms and subsequently to activate the immune system [274]. When macrophages recognize the antigen, the assembly of the protein complex of cytochrome I from the ETC is prevented, and mitochondrial respiration on complexes I and II is increased under the influence of phagosomal NOX and ROS-dependent tyrosine kinase Fgr. This process leads in increase in the ROS concentration [275]. The presence of ROS leads to the activation of MAPK, extracellular signal-regulated kinases (ERK), c-Jun N-terminal kinases (JNK) and NF-kB, which are key factors in the secretion of chemokines and pro-inflammatory cytokines (TNF-α, IL-1β, IL-6, IFN-γ) [276,277], as shown in Figure 5. Likewise, in this process of T-cell activation, NOX is also involved, which is activated by reactive species generated at the mitochondrial level, in order to maintain the proper ROS levels for this process [278]. Therefore, the presence of ROS is necessary for the destruction of the bacteria, which is contrast to the situation in chronic granulomatosis disease, in which ROS cannot be generated due to a genetic defect of NOX [279,280].

A certain basal level of ROS is essential for the proper functioning of the immune system, given that the destruction of pathogenic microorganisms by leukocytes is based on the generation of reactive species [281,282,283]. As previously mentioned in this review, ROS are intra- and intercellular communication pathways, along with MAPK, which uses these reactive species to generate cytokines and activate the immune system. Moreover, the activation of T lymphocytes and macrophages requires limited amounts of $O_{2}^{*-}$ and H_2_O_2_; thus, neutralizing these physiological levels would lead to adverse effects on the immune system. Nevertheless, it is necessary that a balance of this oxidative stress is maintained since excess ROS can jeopardize the viability of T-cells. Thus, T lymphocyte functionality is dependent on ROS generation, as well as on the reduction of these species. Activated T-cells limit the permeability of the membrane and prevent ROS migration through the cytoplasm. At the same time, it has been discovered that antigen-presenting dendritic cells (APCs) release Cys in contact with T-cells. Cys is then captured and used by T-cells for GSH synthesis in order to protect them from excess ROS. Based on these considerations, antioxidant systems, as well as ROS, are essential for the proper functioning of the adaptive immune system mediated by T-cells [284,285].

Another area affected by the excessive use of antioxidant compounds is the biochemical one, in which interference with different metabolic pathways can occur. Given that the reactions of the Krebs cycle or in the ETC are redox phenomena, maintaining a long-term reduction status can have negative effects, such as the slowing down of metabolism, the accumulation of intermediates, and decreased ATP synthesis, with all these reactions being interconnected [286,287]. Another mechanism to control free radicals generated by ROS is based on heat shock proteins (HSPs) [288]. These proteins have properties to correct the misfolding of polypeptides and to promote the degradation of altered peptides. Under physiological conditions, the expression of HSPs is induced by various factors, including ROS, but the irresponsible use of antioxidant compounds can have negative effects on the activity and synthesis of HSPs [289,290,291].

The administration of antioxidants in viral infections has provided only evasive data, because in some studies, the benefits are not highlighted, and the antioxidants can favor the progression of the infection [292]. In the antioxidant therapeutic approach in cancer cells, the level of ROS must be considered because it can favor the situation in which, in the absence of a proper level of ROS, the cancer therapy can promote carcinogenesis via NF-kB, HIFs, and MAPK activation [27]. Paradoxically, cancer cells induce their own antioxidant systems with the purpose of reducing excess ROS, which would be detrimental through cytotoxic effects. In this context, there are two therapeutic approaches, one of which is based on antioxidant administration, which could reduce ROS levels required for the proliferation of cancer cells, making them susceptible to cellular apoptosis. However, this approach has raised numerous questions on this topic, and the results have been evasive [293].

The second approach refers to inducing oxidative stress to reach levels that cannot be neutralized by cancer cells. The therapy can be performed directly with therapeutic agents that promote ROS generation (radio- and chemotherapy) or indirectly, with agents that inhibit the antioxidant systems [294]. In this manner, the existence of the tumor cells depends on ROS levels; moderate levels activate NF-kB and promote survival mechanisms, whereas excessive levels degrade the cellular organelles, specifically the mitochondria, and alter the DNA and ARN molecules, favoring the initiation of apoptotic reactions [295].

For this reason, it is important to acknowledge the oxidative status of each individual, as antioxidants should not be administered to individuals without a proper immune response (see Table 2). However, a decrease in the activity of the immune system requires the antioxidant therapy approach.

## 5. Conclusions

The data presented in the literature suggest that ROS and RNS negatively influence the human body, but that this effect depends to a great extent on the individual’s oxidative status, as ROS are also involved in growth, differentiation, proliferation, and the processes of apoptosis and autophagy, which are highly important for maintaining cellular homeostasis. Medium-intensity aerobic exercise is responsible for generating ROS at levels that are beneficial and have the ability to induce counterregulatory and adaptation mechanisms, such as PGC-1α. In striated muscles, adaptation mechanisms are induced by exposure to ROS and RNS produced during resistance training and are mainly mediated through NF-kB and PGC-1α. With age, the incidence of neurodegenerative diseases increases, but a well-balanced CR seems to diminish this trend. Animal studies confirm the hormesis theory as CR is a form of stress itself, inducing adaptation mechanisms in the brain through the endocrine system. Thus, the expression of neurotrophic factors is dependent on CR and neurogenesis is favored.

In fact, the present article discusses an evolution paradox, the transition from anaerobic to aerobic metabolism and how, during evolution, the body manages to use ROS generated from aerobic metabolism, which is harmful, in purposeful ways to ensure adaptability, homeostasis, and cell signaling. Another interesting fact to emphasize is the duality of these reactive species, which are harmful yet indispensable for living.

In addition, given the delicate regulation mechanisms that dictate the generation and elimination rates, the duality of ROS (beneficial and negative effects) must be considered in therapy. In case of antitumor therapies, there are some limitations, i.e., toxic and/or immunosuppressive effects of agents generating ROS. Therefore, the dosage of anti/pro-oxidant agents has to target the maintenance of redox signaling capacity between the unaffected cells.

The biological dose–response curve in oxidative stress is unpredictable as reactive species are clearly responsible for cellular degradation, whereas antioxidant therapies can alleviate senescence by maintaining redox balance. Nevertheless, excessive doses of the latter can modify the redox balance of the cell, leading to a negative outcome. In order to avoid redox lesions, a balanced diet and moderate-intensity physical exercises are recommended to be performed regularly, especially for non-trained individuals.

The implication of ROS in many different processes, such as cell proliferation and differentiation, signal transduction, apoptosis, and autophagy, is not surprising, considering that these species have been present since the appearance of aerobic organisms, and have become indispensable for the abovementioned processes. It is supposed that cellular apoptosis does not occur directly due to oxidative stress, but due to ROS species that activate the physiological processes of apoptosis. It has also been observed that the reduction of ROS levels below a certain threshold would impede cell proliferation and differentiation and the immune response. Based on this, we can infer the essential role of the presence of ROS in the homeostasis of the body.

For this reason, it is important to practice responsible behavior when using antioxidant compounds, particularly for individuals who are known to have an affected antioxidant status.

## Figures and Tables

**Table 1:** Other effects related to excessive supplementation with different antioxidants.

Antioxidant Agent (Large Doses That Exceed Recommended Daily Doses)	Other Effects	Ref.
Vitamin A	Birth defects/teratogenicity	[231,232,233]
	Increases triglycerides, total cholesterol, decreases high-density lipoprotein cholesterol (HDL)	[234,235]
	Hip fracture, osteoporosis?	[236,237,238]
β-carotene	Lung cancer in smokers	[239]
Vitamin C	Kidney stones	[240,241]
	Cardiovascular disease?	[242,243,244]
	Free radical generation?	[12,245]
Vitamin E	Prostate cancer	[246]
	Increased hemorrhagic stroke?	[233]
	Decreased birth weight	[247]
	Lung cancer in smokers	[239,248]
Selenium	Prostate cancer	[249]
	Hashimoto’s thyroiditis?	[250,251]
Zinc	Impaired lipoprotein metabolism	[252]
	Impaired immune response	

? indicates evasive/inconclusive data.

**Table 2:** Positive and negative effects related to the generation of oxidative stress on organs, systems, diseases, and physiological conditions.

	Positive Effects	Ref.	Negative Effects	Ref.
Muscle	Improves antioxidant defense	[296,297,298,299]	Contractile dysfunction Muscle atrophy and weakness	[300,301]
	Adaptive response (muscle fiber type adaptation)			
	Increases the number of mitochondria			
Brain	Synaptic plasticity	[302,303,304,305]	Neuropsychiatric disorder Neuron apoptosis Neurotoxicity Neurodegenerative diseases	[306,307,308,309,310]
	Hippocampus-independent memory formation			
Immune system	Increases immune system activity ↗NF-KB ↗IL-1β ↗TNF-α	[311]	Rheumatoid arthritis Respiratory diseases Periodontitis Chronic kidney disease Gastro-intestinal autoimmune disease Multiple sclerosis Fibromyalgia	[312,313,314,315]
Vascular system	Vasodilation Regulates blood pressure Angiogenesis Wound healing Granulation tissue formation	[140,316,317]	Hypertension Atherosclerosis Ischemia reperfusion	[318,319,320]
Cancer	Cycle arrest Apoptosis Inhibits metastasis	[321,322,323,324,325,326]	Carcinogenesis Metastasis Therapy resistance	[77,327,328]
Aging	Muscle remodeling Neuronal activity Preservation	[12,159,197,212,329]	Sarcopenia Muscular dystrophy DNA damage Telomere shortening Neurodegenerative diseases	[202,330,331,332,333,334,335]
